# Truffle fungi enriched in cattle-grazed native pecan groves under NPK fertilization

**DOI:** 10.1093/hr/uhaf308

**Published:** 2025-11-06

**Authors:** Amandeep Kaur, Wei Ren, Tingying Xu, Niels Maness, Shiping Deng, Jahanifard Mohadeseh, Zobair Rahman Biswas, Harpreet Singh, Lu Zhang

**Affiliations:** Department of Horticulture and Landscape Architecture, Oklahoma State University, Stillwater, OK, USA; Boone Pickens School of Geology, Oklahoma State University, Stillwater, OK, USA; Boone Pickens School of Geology, Oklahoma State University, Stillwater, OK, USA; Department of Horticulture and Landscape Architecture, Oklahoma State University, Stillwater, OK, USA; Department of Plant and Soil Sciences, Oklahoma State University, Stillwater, OK, USA; Department of Horticulture and Landscape Architecture, Oklahoma State University, Stillwater, OK, USA; Department of Horticulture and Landscape Architecture, Oklahoma State University, Stillwater, OK, USA; Department of Plant and Soil Sciences, Oklahoma State University, Stillwater, OK, USA; Department of Horticulture and Landscape Architecture, Oklahoma State University, Stillwater, OK, USA

Dear Editor,

Pecan (*Carya illinoinensis* [Wangenh.] K. Koch) is a major tree nut crop native to North America, with Oklahoma contributing nearly 80% of US native and seedling pecan production in 2023 (USDA, 2024). Native pecan groves typically lack structured orchard practices such as fertilization, irrigation, or herbicide application. Instead, they depend on natural nutrient cycling. Among rhizosphere microbes, ectomycorrhizal (ECM) fungi, including truffle-forming Tuber species—a group known for their ecological and commercial value, form mutualistic associations with pecan roots and, in some species, produce edible truffles [1, 2]. Fertilization is known to restructure microbial communities, sometimes enhancing certain ECM taxa while suppressing others [3]. However, the response of ECM fungi, particularly Tuber, to fertilization remains largely unexplored in native pecan groves. Here, we report evidence that cattle grazing combined with NPK fertilization promotes the enrichment of Tuber species, including the pecan truffle (*Tuber lyonii*), in Oklahoma native pecan groves. We analyzed fungal communities from roots and rhizosphere soils of two native pecan groves with contrasting management: Grove A, incorporated cattle grazing while Grove B incorporated hay production but not grazing. In each grove, two fertilizer treatments (Full NPK consisting of 300lbs/acre fertilizer in ratio of 11 N:27P:27 K versus No NPK fertilizer) were utilized, and roots and rhizosphere soils were sampled prior to fertilization in February and after fertilization in May and September 2023. Total DNA was extracted and sequenced (Illumina MiSeq). The sequences were processed with Mothur (v1.48) [4] and R (v4.4.1). Relative abundances of fungal taxa were quantified at genus and species levels, with emphasis on ectomycorrhiza, particularly the genus Tuber. Fungal communities showed strong variation across groves, fertilization, and season. In Grove A (cattle grazing), Tuber and Russula dominated roots, while Grove B (hay) roots were dominated by Inocybe and Russula**.** In Grove A roots in September under Full NPK, relative abundance of Tuber (~20%) was higher compared to No NPK (~9%) (Fig. 1A). Although Tuber abundance was lower in roots from Grove B, NPK fertilization increased it is abundance in the rhizosphere soil (Fig. 1B). Fertilization boosted Tuber abundance in both groves, but the effect was most pronounced in grazed systems, suggesting that grove legacy interacts with nutrient inputs to shape ECM composition (Fig. 1C). Species-level resolution revealed five Tuber taxa: *T. lyonii*, *T. walkeri*, *T. floridanum*, *T. brennemanii*, and *T. mexiusanum* (Fig. 1A). Among these, *T. walkeri* was the most widespread across groves, while *T. lyonii* was concentrated in Grove A roots and increased under Full NPK during September, aligning with its typical fruiting season. *T. floridanum* was also favored in Grove A, while *T. mexiusanum* was enriched in Grove B under No NPK, indicating their sensitivity to fertilization. *T. brennemanii* appeared more tolerant of nutrient additions, persisting with or without NPK fertilization. These species-specific responses demonstrate ecological filtering within native pecan systems. Functional guild analysis confirmed ECM fungi as the dominant group in both native pecan roots and soils (Fig. 1C), consistent with the well-established role of ECM associations with improved pecan roots [4]. In roots, ECM abundance was only slightly higher under Full NPK (41%) than No NPK (37%) (nonsignificant). Soil showed significant variation, overall, with higher ECM in Grove B and Full NPK treatment. Seasonal variation was also evident: in Grove A, *T. lyonii* and *T. walkeri* peaked in September, coinciding with late-season carbon allocation. This suggests that fertilizer inputs, grazing, and seasonal host physiology jointly influenced truffle colonization dynamics.

**Figure 1:**
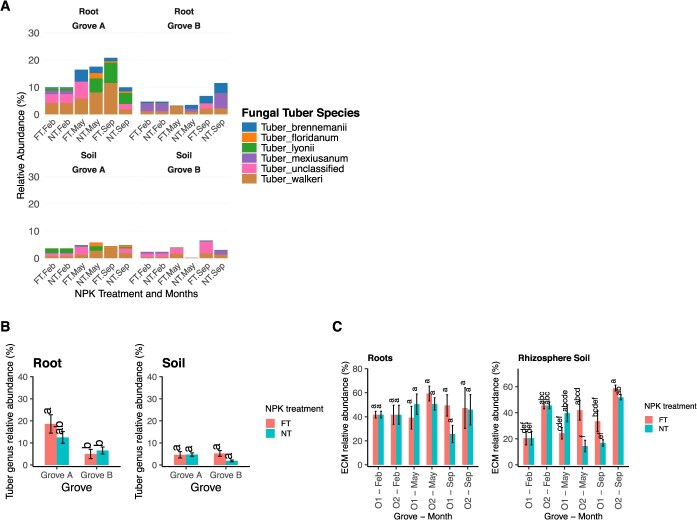
Relative abundance (%) of Tuber Species (A), Tuber genus (B), and ectomycorrhiza (C) in root and rhizosphere soil of native Pecan Groves A(O1) and B(O2) under nitrogen treatments. FT = Full NPK, NT = No NPK; Feb, May, Sept = sampling months. Data are means±SE (*N* = 5 per treatment per grove; 20 root and 20 soil samples total). Different lowercase letters indicate significant differences by Tukey’s HSD test (*P* < 0.05) (After ANOVA).

The economic potential of these findings could be significant. *T. lyonii*, the pecan truffle, is already harvested in improved pecan orchards [1], and its enrichment under grazing and fertilization suggests native groves could also be managed as dual-use systems. This shift would allow native groves, traditionally valued for cattle and nut production, to be managed for cattle, nuts, and truffles. Such co-production could diversify growers’ income and align native pecan systems with European truffle–orchard models. Management appears species-specific: *T. lyonii* and *T. walkeri* benefit from NPK, while *T. mexiusanum* persists in unfertilized refugia. Tailoring fertilization and grazing could optimize both truffle biodiversity and pecan yield. Our findings suggest that under the right grove management fertilization can enhance desirable truffle taxa, consistent with recent reports that moderate nitrogen inputs may favor symbiotrophic fungi [3]. This expands our understanding of nutrient–fungi–host interactions and demonstrates how management legacies condition microbial responses. Fostering ECM fungi, particularly truffles, may enhance both ecological functions, such as nutrient acquisition, stress resilience, and economic outcomes through potential co-harvest of nuts and truffles. In conclusion, we provide the first report of species-specific Tuber enrichment in native pecan groves, indicating that cattle grazing and NPK fertilization may foster conditions favorable for pecan truffle production. Grove A, the grazed system, supported higher *T. lyonii* and *T. walkeri* abundance, particularly in fertilized roots during September. These results highlight the potential for integrating truffle co-cultivation into native pecan management, representing a novel horticultural innovation. Future research should confirm truffle fruiting in these groves, evaluate inoculation strategies, and quantify economic returns from dual-use pecan–truffle systems.

## Data Availability

All data supporting the findings of this study are available in the article.
